# Exploring the top 30 drugs associated with drug-induced constipation based on the FDA adverse event reporting system

**DOI:** 10.3389/fphar.2024.1443555

**Published:** 2024-09-02

**Authors:** Wenwen Li, Cuncheng Liu, Zhongyi Zhang, Zhikai Cai, Tailong Lv, Ruiyuan Zhang, Yaoyao Zuo, Shouqiang Chen

**Affiliations:** ^1^ Second School of Clinical Medicine, Shandong University of Traditional Chinese Medicine, Jinan, China; ^2^ Department of Neonatology, Weifang Traditional Chinese Hospital, Weifang, China

**Keywords:** adverse drug events, constipation, drug-induced, signal detection, United States food and drug administration adverse event reporting system

## Abstract

**Objective:**

This project aims to identify the top 30 drugs most commonly associated with constipation and their signal values within the FDA Adverse Event Reporting System database.

**Methods:**

We extracted adverse drug events (ADEs) related to constipation from the FAERS database spanning from January 1, 2004, to September 30, 2023. We compiled the 30 most frequently reported drugs based on the frequency of constipation events. We employed signal detection methodologies to ascertain whether these drugs elicited significant signals, including reporting odds ratio, proportional reporting ratio, multi-item gamma Poisson shrinker, and information component given by the Bayesian confidence propagation neural network. Furthermore, we conducted a time-to-onset (TTO) analysis for drugs generating significant signals using the medians, quartiles, and the Weibull shape parameter test.

**Results:**

We extracted a total of 50, 659, 288 ADEs, among which 169,897 (0.34%) were related to constipation. We selected and ranked the top 30 drugs. The drug with the highest ranking was lenalidomide (7,730 cases, 4.55%), with the most prevalent drug class being antineoplastic and immunomodulating agents. Signal detection was performed for the 30 drugs, with constipation risk signals identified for 26 of them. Among the 26 drugs, 22 exhibited constipation signals consistent with those listed on the FDA-approved drug labels. However, four drugs (orlistat, nintedanib, palbociclib, and dimethyl fumarate) presented an unexpected risk of constipation. Ranked by signal values, sevelamer carbonate emerged as the drug with the strongest risk signal [reporting odds ratio (95% CI): 115.51 (110.14, 121.15); PRR (χ^2^): 83.78 (191,709.73); EBGM (EB05): 82.63 (79.4); IC (IC025): 6.37 (4.70)]. A TTO analysis was conducted for the 26 drugs that generated risk signals, revealing that all drugs exhibited an early failure type. The median TTO for orlistat was 3 days, the shortest of all the drugs, while the median TTO for clozapine was 1,065 days, the longest of all the drugs.

**Conclusion:**

Our study provides a list of drugs potentially associated with drug-induced constipation (DIC). This could potentially inform clinicians about some alternative medications to consider when managing secondary causes of constipation or caring for patients prone to DIC, thereby reducing the incidence and mortality associated with DIC.

## 1 Introduction

Constipation is a prevalent gastrointestinal issue characterized by unsatisfactory bowel movements, often presenting as decreased defecation frequency, difficulty passing stools, or a combination thereof (Feng et al., 2024). As reported, the prevalence of constipation varies from 4.1% to 25.6%, rendering it a significant public health concern (Turawa et al., 2020). Constipation is classified into primary and secondary forms, with medication being an essential factor contributing to secondary constipation (Scott et al., 2021). Studies have indicated that drug-induced constipation (DIC) accounts for approximately 11% of all cases of treated constipation (Karasawa et al., 2024). Another study has shown that constipation is a common symptom reported by patients during clinical medication reviews for drug-related assessments (Schoenmakers et al., 2017). It can be seen that DIC is not only common in patients with constipation, but also in patients who report adverse drug reactions (ADRs). However, this phenomenon has not received sufficient attention, and DIC is poorly recognized, and its management is neglected. In the course of long-term treatment, DIC may lead to a decline in patients’ compliance, which affects the treatment effect of many diseases and the long-term prognosis of patients. For example, potassium binders are commonly used to treat hyperkalemia, and one of its serious side effects is constipation. Hyperkalemia is associated with mortality and morbidity, especially in elderly patients with heart failure (HF) and/or chronic kidney disease (CKD) (Sarwar et al., 2016; Seliger, 2019; Hida et al., 2023). The presence of constipation can aggravate HF and CKD, leading to further disease progression (Sumida et al., 2017; Ishiyama et al., 2019). However, healthcare professionals may not be aware of the risks of constipation. In cancer patients, DIC can lead to a decline in quality of life and hinder optimal pain management, resulting in severe psychosocial distress for patients (Dzierżanowski and Mercadante, 2022). In addition, despite studies reporting a higher morbidity and mortality of constipation caused by antipsychotic medications, especially clozapine, less attention has been paid to this issue in clinical practice (Chen and Hsieh, 2018; Xu et al., 2021).

In previous studies, a variety of classes of medications associated with constipation have been identified, including antipsychotics (Xu et al., 2021), antidepressants (Jeong et al., 2022), opioids (Liu and Brenner, 2022), iron supplementation (Bumrungpert et al., 2022), and antineoplastic agents (Calsina-Berna et al., 2023). However, most of this literature is specific to a particular class of drugs and lacks systematic summaries and large-scale studies of constipation caused by specific medications. In recent years, the study of large datasets in the medical field, especially the analysis of spontaneous adverse drug event (ADE) reporting databases, has become a hot research topic (Musialowicz et al., 2023; Sharma et al., 2023; Javed and Kumar, 2024). The United States Food and Drug Administration (FDA) Adverse Event Reporting System (FAERS) is the world’s largest database of spontaneous ADE reports, updated quarterly, which can be used to analyze drug-adverse event associations and plays a critical role in post-market drug surveillance (Sharma and Kumar, 2022; Jain et al., 2023). This study was to extract reports of DIC using the FAERS database, analyze and identify the top 30 most common drugs associated with DIC, and calculate their risk signals to provide references for the clinical safety of drug use.

## 2 Methods

### 2.1 Data sources

The FDA processes the collected adverse reaction data electronically and publishes them quarterly online (https://fis.fda.gov/extensions/FPD-QDE-FAERS/FPD-QDE-FAERS.html) in formats such as ASCII and XML, which can be freely downloaded. The downloaded adverse event data were organized to obtain seven subfiles from Q1 2004 to Q3 2023, namely, DEMO (Patient Demographic and Administrative Info), DRUG (Drug/Biologic Info), REAC (MedDRA Terms for Adverse Event), OUTC (Patient Outcomes), RPSR (Report Sources), THER (Drug Therapy Start/End Dates), and INDI (MedDRA Terms for Diagnoses/Indications). ADEs were standardized using the preferred term (PT) from the Medical Dictionary for Regulatory Activities (MedDRA) Dictionary (Version 24.0) to ensure consistency of terminology.

### 2.2 Identification of target data

The number of constipation reports and the PRIMARYID codes were identified based on the PT “constipation” in the REAC files. Subsequently, the PRIMARYID was identified in the DEMO files, and duplicate reports were removed to determine the number of constipation cases. Information on AGE, SEX, and REPORTER_COUNTRY was then extracted. The drug role is limited to primary suspect. Drugs with “secondary suspect”, “concomitant drug”, and “interacting drug” were excluded because of more significant uncertainty about the association between these drugs and reported adverse events.

### 2.3 Statistical analysis

Firstly, descriptive analyses were used to summarize the reported yearly and quarterly distributions and clinical characteristics of constipation cases (including patients’ age, sex, and reporting country).

Secondly, we listed the top 30 drugs most commonly associated with reported constipation events and classified them according to the Anatomical Therapeutic Chemical (ATC) classification system (https://www.who.int/tools/atc-ddd-toolkit/atc-classification). We also calculated the percentage of constipation reports among all ADEs for these medications annually. The 30 drugs were then subjected to signal detection. Signal detection used the reporting odds ratio (ROR) (van Puijenbroek et al., 2002), the proportional reporting ratio (PRR) (van Puijenbroek et al., 2002), the multi-item gamma Poisson shrinker (MGPS) (Szarfman et al., 2002), and the information component (IC) given by Bayesian confidence propagation neural network (BCPNN) (Bate et al., 1998; Bate et al., 2002). All the methods above were statistically analyzed based on the signal detection 2 × 2 contingency table parameters presented in Supplementary Table S1. The formulas for each algorithm and the criteria for signal generation are detailed in Supplementary Table S2. To maximize the identification of potential correlations between drugs and constipation, we considered the generation of a valid signal when the selected drug signal met the criteria of any of the applied algorithms, indicating a potential association between the two.

Finally, we analyzed the adverse reaction onset time for drugs that generated valid signals. The time-to-onset (TTO) of adverse events is defined as the time interval between the occurrence date of adverse events (EVENT_DT) and the start date of drug use (START_DT) (Wu et al., 2022). To ensure the accuracy of calculations reports with input errors (EVENT_DT preceding START_DT), inaccurate date entries, and missing specific data were excluded (Shu et al., 2022). We employed the medians, quartiles, and the Weibull shape parameter (WSP) test to assess TTO (Kinoshita et al., 2020; Shu et al., 2022). Data analysis and visualization were conducted using R 4.2.2, Microsoft Excel 2016, and an online platform (https://www.bioinformatics.com.cn) (Tang et al., 2023).

## 3 Results

### 3.1 Basic characteristics of constipation-related ADE reports

**FIGURE 1:**
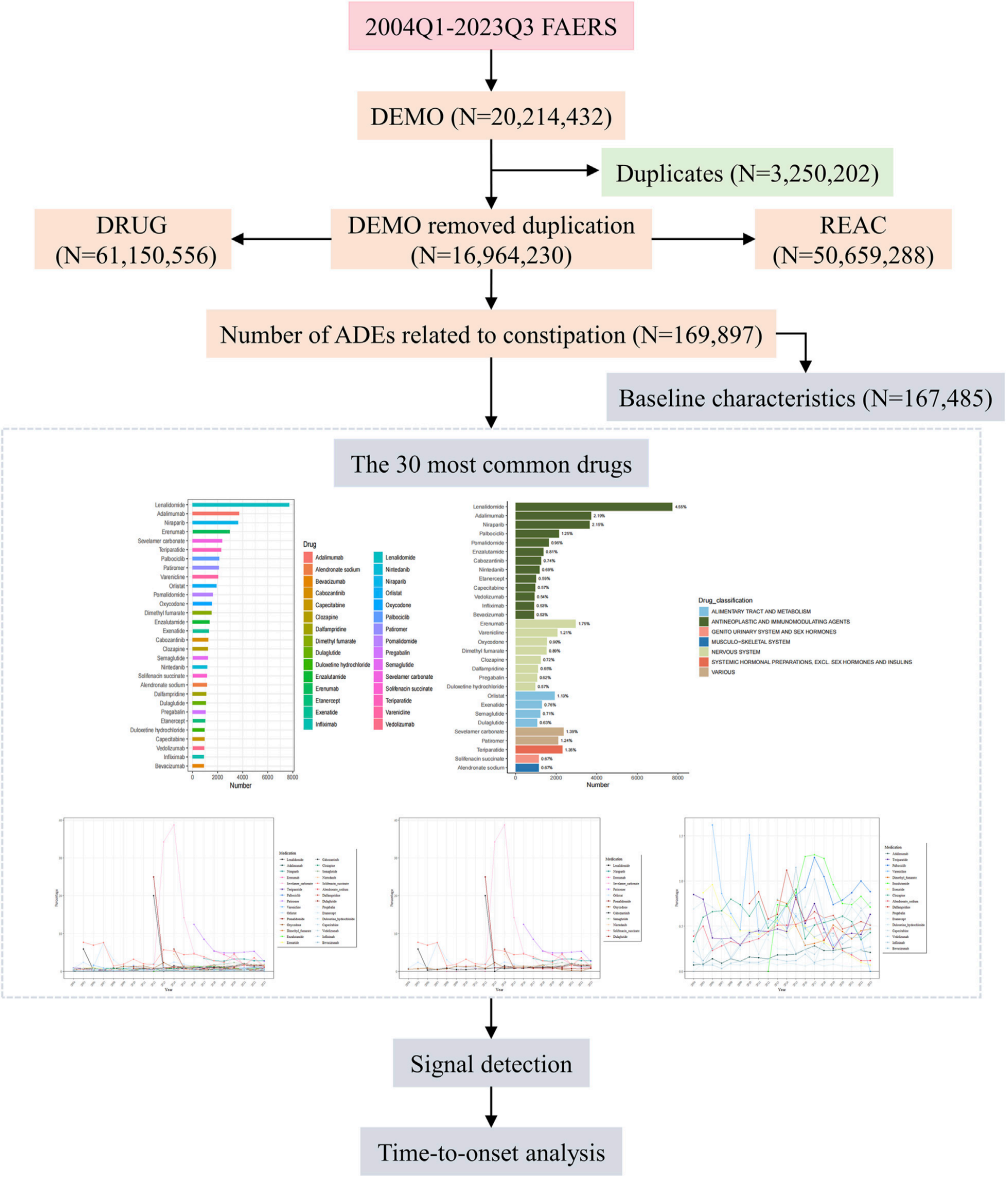
Flow chart for identification of constipation reports of suspected ADEs. ADEs, Adverse drug events; FAERS, United States Food and Drug Administration Adverse Event Reporting System.

Data from 79 quarters from the first quarter of 2004 to the third quarter of 2023 were retrieved from the FAERS database, yielding 50, 659, 288 ADEs, among which 169,897 (0.34%) were related to DIC. The entire research process is illustrated in Figure 1.

**FIGURE 2:**
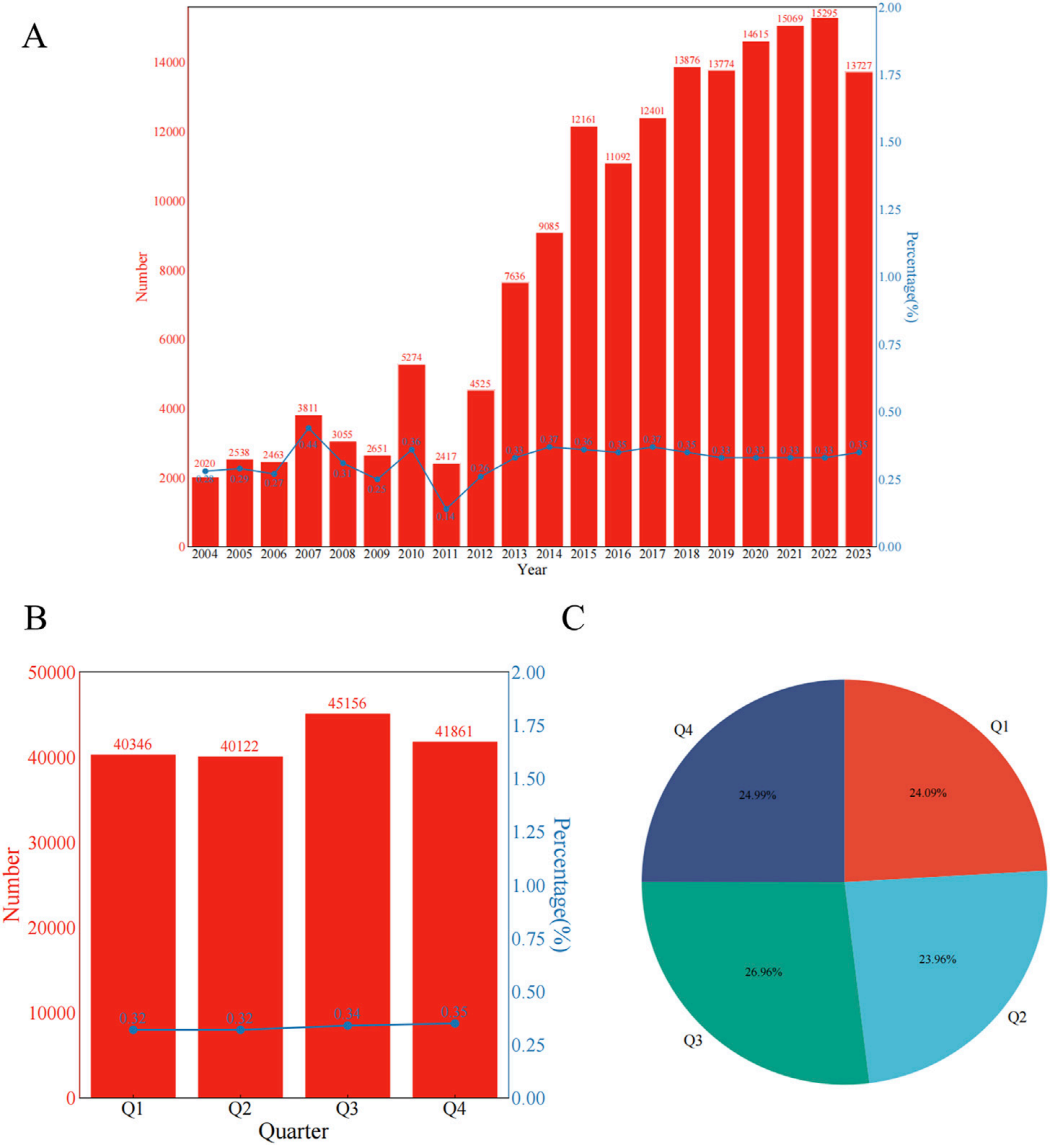
From Q1 2004 to Q3 2023, annual and quarterly characteristics of constipation event cases were reported **(A)** Number of reported cases of constipation per year and percentage of cases of constipation per year among all adverse drug events per year **(B)** Number of reported cases of constipation per quarter and percentage of cases of constipation per quarter among all adverse drug events per quarter **(C)** The percentage of constipation cases reported each quarter relative to all constipation cases.

A total of 167,485 patients were included in the study. Since the establishment of the database in 2004, ADEs related to constipation have been reported annually. Figure 2A illustrates the annual number of reported constipation cases and the yearly percentage of constipation cases among all ADEs. Figure 2B shows the quarterly number of constipation cases and the percentage of quarterly constipation cases among all ADEs each quarter. Figure 2C shows the percentage of constipation cases reported each quarter relative to the total number of constipation cases. Furthermore, we reported the characteristics of the 167,485 patients, including age, sex, and reporting country. Detailed information can be found in Table 1.

**TABLE 1:** General characteristic of patients included in study.

Characteristics	Cases, n (%)
Total	167,485 (100)
Age	
<18 years	2,988 (1.8)
≥18 and <40 years	10,156 (6.1)
≥40 and <65 years	41,501 (24.8)
≥65 years	47,130 (28.1)
Unknown	65,710 (39.2)
Sex	
Male	55,738 (33.3)
Female	94,827 (56.6)
Unknown	16,920 (10.1)
Reported Countries (Top five)	
United States	119,187 (71.2)
Canada	9,608 (5.7)
Great Britain	6,548 (3.9)
Japan	4,092 (2.4)
Germany	3,685 (2.2)

### 3.2 The 30 most common drugs

**FIGURE 3:**
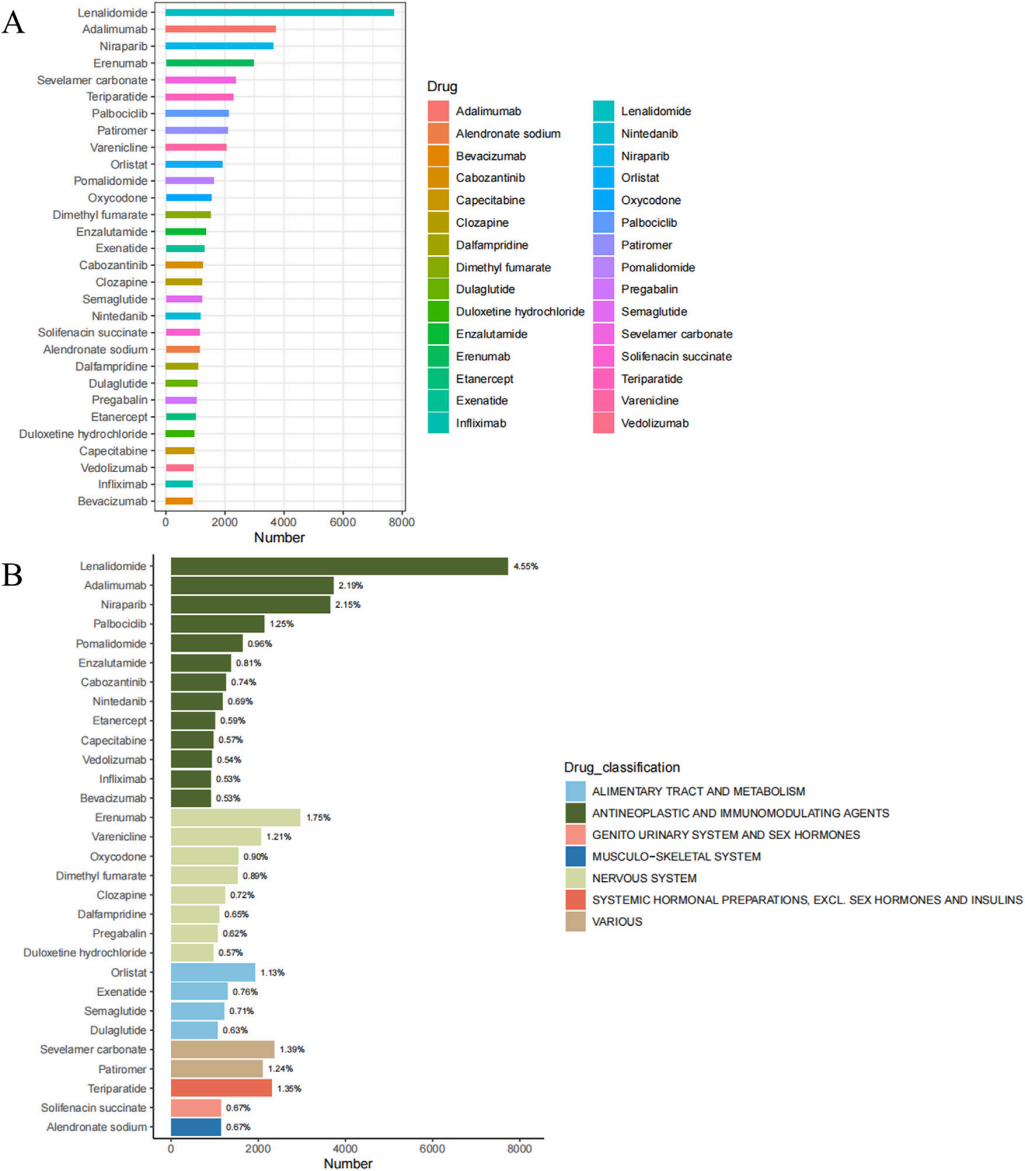
Top 30 medications with the most constipation cases in the FAERS database, January 1, 2004 to September 30, 2023 **(A)** Number of cases of the 30 most common drugs **(B)** The drug categories of the 30 most common drugs and the percentage of total constipation events associated with each drug.

Based on the counts of constipation-related ADEs, we identified the top 30 drugs (Table 2; Figure 3A). These include lenalidomide (7,730 cases, 4.55%), adalimumab (3,720 cases, 2.19%), niraparib (3,651 cases, 2.15%), erenumab (2,966 cases, 1.75%), sevelamer carbonate (2,369 cases, 1.39%), teriparatide (2,300 cases, 1.35%), palbociclib (2,127 cases, 1.25%), patiromer (2,100 cases, 1.24%), varenicline (2,059 cases, 1.21%), orlistat (1,918 cases, 1.13%), pomalidomide (1,628 cases, 0.96%), oxycodone (1,537 cases, 0.90%), dimethyl fumarate (1,517 cases, 0.89%), enzalutamide (1,368 cases, 0.81%), exenatide (1,294 cases, 0.76%), cabozantinib (1,260 cases, 0.74%), clozapine (1,227 cases, 0.72%), semaglutide (1,214 cases, 0.71%), nintedanib (1,177 cases, 0.69%), solifenacin succinate (1,135 cases, 0.67%), alendronate sodium (1,135 cases, 0.67%), dalfampridine (1,099 cases, 0.65%), dulaglutide (1,066 cases, 0.63%), pregabalin (1,054 cases, 0.62%), etanercept (1,007 cases, 0.59%), duloxetine hydrochloride (963 cases, 0.57%), capecitabine (963 cases, 0.57%), vedolizumab (922 cases, 0.54%), infliximab (905 cases, 0.53%), and bevacizumab (904 cases, 0.53%). We categorized the 30 drugs (Table 2; Figure 3B) and found that antineoplastic and immunomodulating agents (lenalidomide, adalimumab, niraparib, palbociclib, pomalidomide, enzalutamide, cabozantinib, nintedanib, etanercept, capecitabine, vedolizumab, infliximab, and bevacizumab) were the most common in cases of constipation. These were followed by nervous system (erenumab, varenicline, oxycodone, dimethyl fumarate, clozapine, dalfampridine, pregabalin, and duloxetine hydrochloride), alimentary tract and metabolism (orlistat, exenatide, semaglutide, and dulaglutide), and various (sevelamer carbonate and patiromer). Additionally, systemic hormonal preparations, excl. Sex hormones and insulins (teriparatide), genito urinary system and sex hormones (solifenacin succinate), and musculo-skeletal system (alendronate sodium) were also reported.

**TABLE 2:** Top 30 constipation-related medications by frequency in the FAERS database, January 1, 2004 to September 30, 2023.

Ranking	Medication	Frequency	Percentage (%)	Classification
1	Lenalidomide	7,730	4.55	L
2	Adalimumab	3,720	2.19	L
3	Niraparib	3,651	2.15	L
4	Erenumab	2,966	1.75	N
5	Sevelamer carbonate	2,369	1.39	V
6	Teriparatide	2,300	1.35	H
7	Palbociclib	2,127	1.25	L
8	Patiromer	2,100	1.24	V
9	Varenicline	2059	1.21	N
10	Orlistat	1918	1.13	A
11	Pomalidomide	1,628	0.96	L
12	Oxycodone	1,537	0.90	N
13	Dimethyl fumarate	1,517	0.89	N
14	Enzalutamide	1,368	0.81	L
15	Exenatide	1,294	0.76	A
16	Cabozantinib	1,260	0.74	L
17	Clozapine	1,227	0.72	N
18	Semaglutide	1,214	0.71	A
19	Nintedanib	1,177	0.69	L
20	Solifenacin succinate	1,135	0.67	G
21	Alendronate sodium	1,135	0.67	M
22	Dalfampridine	1,099	0.65	N
23	Dulaglutide	1,066	0.63	A
24	Pregabalin	1,054	0.62	N
25	Etanercept	1,007	0.59	L
26	Duloxetine hydrochloride	963	0.57	L
27	Capecitabine	963	0.57	N
28	Vedolizumab	922	0.54	L
29	Infliximab	905	0.53	L
30	Bevacizumab	904	0.53	L

A, alimentary tract and metabolism; FAERS, United States food and drug administration adverse event reporting system; G, genito urinary system and sex hormones; H, systemic hormonal preparations, excl. Sex hormones and insulins; L, antineoplastic and immunomodulating agents; M, musculo-skeletal system; N, nervous system; V, various.

**FIGURE 4:**
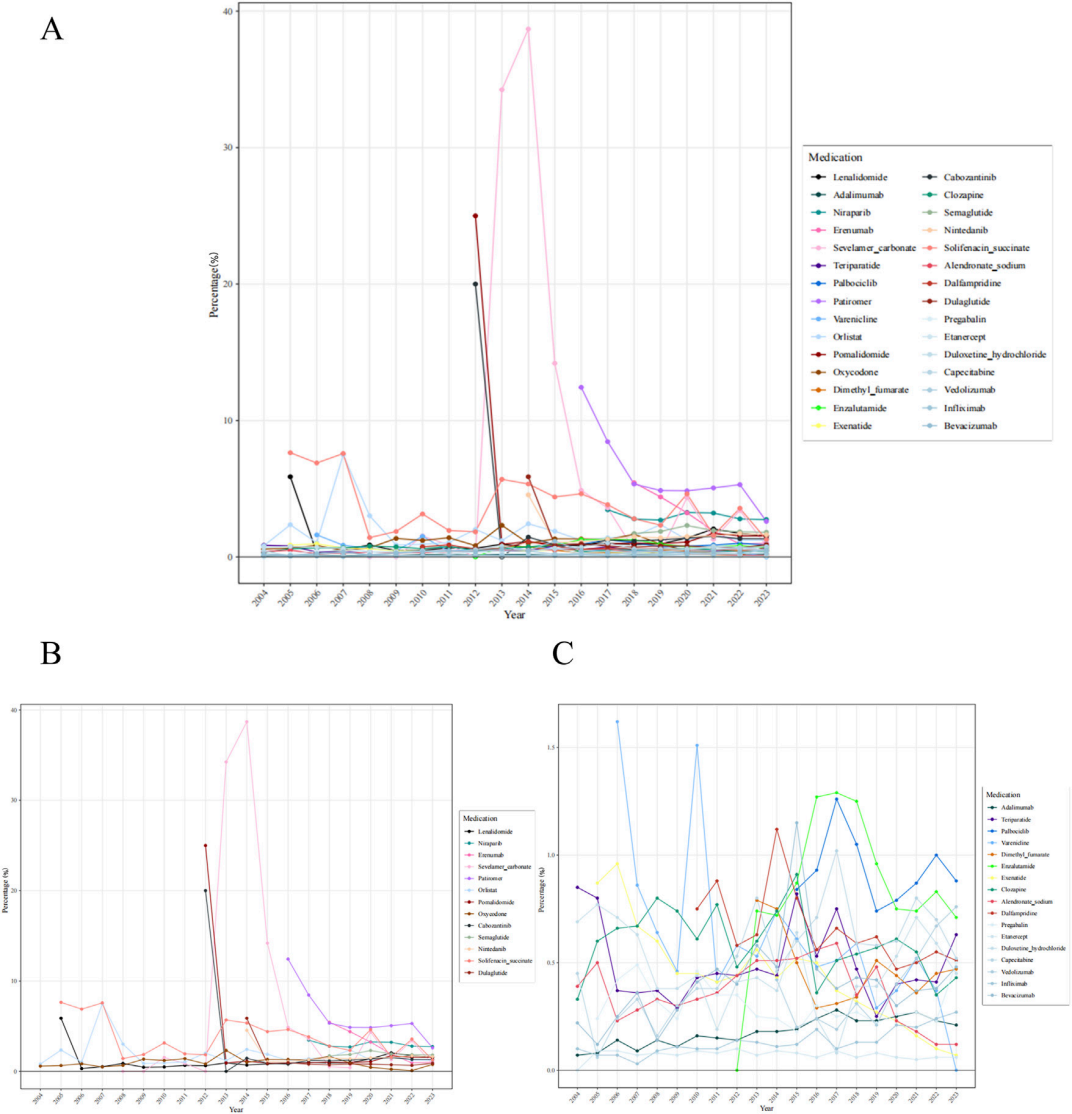
Percentage of constipation reports among adverse drug events for 30 medications annually **(A)** 30 medications **(B)** 13 medications exhibited percentages exceeding 2% in specific years **(C)** 17 medications with a percentage ≤2%.

To mitigate the bias arising from the fact that some drugs have been on the market for many years while others have been introduced more recently and that some drugs may be prescribed more frequently than others, we calculated the percentage of constipation reports for 30 drugs relative to the total number of ADEs reported for each drug in the corresponding year (Figure 4). The results indicate that sevelamer carbonate exhibited the highest percentage among the assessed drugs. Thirteen drugs (sevelamer carbonate, pomalidomide, cabozantinib, patiromer, solifenacin succinate, orlistat, lenalidomide, dulaglutide, erenumab, nintedanib, niraparib, oxycodone, and semaglutide) exhibited percentages exceeding 2% in specific years.

### 3.3 Signal detection

We calculated the signal values for the 30 most common drugs using four methods. The results are presented in Table 3. According to the ROR criteria, 26 of the 30 drugs generated signals. The results are as follows: sevelamer carbonate [ROR (95% CI): 115.51 (110.14, 121.15)], patiromer [ROR (95% CI): 24.90 (23.81, 26.04)], solifenacin succinate [ROR (95% CI): 14.76 (13.91, 15.67)], orlistat [ROR (95% CI): 11.46 (10.94, 11.99)], erenumab [ROR (95% CI): 9.65 (9.30, 10.01)], niraparib [ROR (95% CI): 9.14 (8.84, 9.45)], semaglutide [ROR (95% CI): 5.81 (5.49, 6.15)], nintedanib [ROR (95% CI): 4.56 (4.31, 4.83)], cabozantinib [ROR (95% CI): 4.06 (3.84, 4.29)], lenalidomide [ROR (95% CI): 3.81 (3.72, 3.90)], pomalidomide [ROR (95% CI): 3.71 (3.53, 3.89)], enzalutamide [ROR (95% CI): 3.19 (3.02, 3.36)], varenicline [ROR (95% CI): 2.85 (2.73, 2.98)], palbociclib [ROR (95% CI): 2.82 (2.70, 2.95)], dulaglutide [ROR (95% CI): 2.36 (2.22, 2.50)], teriparatide [ROR (95% CI): 2.00 (1.92, 2.09)], dalfampridine [ROR (95% CI): 1.93 (1.82, 2.05)], capecitabine [ROR (95% CI): 1.90 (1.78, 2.03)], clozapine [ROR (95% CI): 1.71 (1.61, 1.81)], vedolizumab [ROR (95% CI): 1.66 (1.56, 1.78)], exenatide [ROR (95% CI): 1.57 (1.49, 1.66)], dimethyl fumarate [ROR (95% CI): 1.54 (1.46, 1.62)], bevacizumab [ROR (95% CI): 1.51 (1.41, 1.61)], duloxetine hydrochloride [ROR (95% CI): 1.28 (1.20, 1.36)], alendronate sodium [ROR (95% CI): 1.23 (1.16, 1.31)], and oxycodone [ROR (95% CI): 1.07 (1.02, 1.13)]. According to the PRR criteria, 15 drugs generated signals. The results are as follows: sevelamer carbonate [PRR (χ^2^): 83.78 (191,709.73)], patiromer [PRR (χ^2^): 23.07 (43,942.56)], solifenacin succinate [PRR (χ^2^): 14.12 (13,787.01)], orlistat [PRR (χ^2^): 11.07 (17,433.80)], erenumab [PRR (χ^2^): 9.38 (21,897.46)], niraparib [PRR (χ^2^): 8.90 (25,133.71)], semaglutide [PRR (χ^2^): 5.72 (4,707.24)], nintedanib [PRR (χ^2^): 4.51 (3,201.12)], cabozantinib [PRR (χ^2^): 4.02 (2,846.02)], lenalidomide [PRR (χ^2^): 3.78 (15,109.69)], pomalidomide [PRR (χ^2^): 3.67 (3,149.79)], enzalutamide [PRR (χ^2^): 3.16 (2016.09)], varenicline [PRR (χ^2^): 2.84 (2,427.64)], palbociclib [PRR (χ^2^): 2.81 (2,448.58)], and dulaglutide [PRR (χ^2^): 2.34 (819.90)]. According to the MGPS criteria, 15 drugs generated signals. The results are as follows: sevelamer carbonate [EBGM (EB05): 82.63 (79.4)], patiromer [EBGM (EB05): 22.8 (21.96)], solifenacin succinate [EBGM (EB05): 14.03 (13.34)], orlistat [EBGM (EB05): 10.96 (10.55)], erenumab [EBGM (EB05): 9.24 (8.96)], niraparib [EBGM (EB05): 8.73 (8.49)], semaglutide [EBGM (EB05): 5.68 (5.42)], nintedanib [EBGM (EB05): 4.48 (4.27)], cabozantinib [EBGM (EB05): 4 (3.81)], lenalidomide [EBGM (EB05): 3.65 (3.58)], pomalidomide [EBGM (EB05): 3.65 (3.5)], enzalutamide [EBGM (EB05): 3.15 (3.01)], varenicline [EBGM (EB05): 2.81 (2.71)], palbociclib [EBGM (EB05): 2.78 (2.68)], and dulaglutide [EBGM (EB05): 2.34 (2.22)]. According to the BCPNN criteria, 11 drugs generated signals. The results are as follows: sevelamer carbonate [IC (IC025): 6.37 (4.70)], patiromer [IC (IC025): 4.51 (2.84)], solifenacin succinate [IC (IC025): 3.81 (2.14)], orlistat [IC (IC025): 3.45 (1.79)], erenumab [IC (IC025): 3.21 (1.54)], niraparib [IC (IC025): 3.13 (1.46)], semaglutide [IC (IC025): 2.51 (0.84)], nintedanib [IC (IC025): 2.16 (0.50)], cabozantinib [IC (IC025): 2.00 (0.33)], lenalidomide [IC (IC025): 1.87 (0.20)], and pomalidomide [IC (IC025): 1.87 (0.20)]. Of the drugs assessed using any of the algorithmic criteria, 26 were identified as generating valid signals, while four drugs did not exhibit any discernible risk signals. Among the 26 drugs that generated valid signals, the FDA-approved drug labels for 22 drugs (such as sevelamer carbonate, patiromer, solifenacin succinate, erenumab, *etc.*) documented anticipated constipation ADEs. However, constipation risks beyond those anticipated in the labelling were observed for four drugs (orlistat, nintedanib, palbociclib, and dimethyl fumarate).

**TABLE 3:** Signal scores for drug-associated constipation.

Ranking	Medication	ROR (95%Cl)	PRR (χ^2^)	EBGM (EBGM05)	IC (IC025)
1	Sevelamer carbonate	115.51 (110.14, 121.15)*	83.78 (191,709.73)*	82.63 (79.4)*	6.37 (4.70)*
2	Patiromer	24.90 (23.81, 26.04)*	23.07 (43,942.56)*	22.8 (21.96)*	4.51 (2.84)*
3	Solifenacin succinate	14.76 (13.91, 15.67)*	14.12 (13,787.01)*	14.03 (13.34)*	3.81 (2.14)*
4	Orlistat^#^	11.46 (10.94, 11.99)*	11.07 (17,433.80)*	10.96 (10.55)*	3.45 (1.79)*
5	Erenumab	9.65 (9.30, 10.01)*	9.38 (21,897.46)*	9.24 (8.96)*	3.21 (1.54)*
6	Niraparib	9.14 (8.84, 9.45)*	8.90 (25,133.71)*	8.73 (8.49)*	3.13 (1.46)*
7	Semaglutide	5.81 (5.49, 6.15)*	5.72 (4,707.24)*	5.68 (5.42)*	2.51 (0.84)*
8	Nintedanib^#^	4.56 (4.31, 4.83)*	4.51 (3,201.12)*	4.48 (4.27)*	2.16 (0.50)*
9	Cabozantinib	4.06 (3.84, 4.29)*	4.02 (2,846.02)*	4 (3.81)*	2.00 (0.33)*
10	Lenalidomide	3.81 (3.72, 3.90)*	3.78 (15,109.69)*	3.65 (3.58)*	1.87 (0.20)*
11	Pomalidomide	3.71 (3.53, 3.89)*	3.67 (3,149.79)*	3.65 (3.5)*	1.87 (0.20)*
12	Enzalutamide	3.19 (3.02, 3.36)*	3.16 (2016.09)*	3.15 (3.01)*	1.65 (−0.01)
13	Varenicline	2.85 (2.73, 2.98)*	2.84 (2,427.64)*	2.81 (2.71)*	1.49 (−0.17)
14	Palbociclib^#^	2.82 (2.70, 2.95)*	2.81 (2,448.58)*	2.78 (2.68)*	1.48 (−0.19)
15	Dulaglutide	2.36 (2.22, 2.50)*	2.34 (819.90)*	2.34 (2.22)*	1.22 (−0.44)
16	Teriparatide	2.00 (1.92, 2.09)*	1.99 (1,130.24)	1.98 (1.91)	0.99 (−0.68)
17	Dalfampridine	1.93 (1.82, 2.05)*	1.92 (484.70)	1.92 (1.82)	0.94 (−0.73)
18	Capecitabine	1.90 (1.78, 2.03)*	1.90 (406.31)	1.89 (1.79)	0.92 (−0.75)
19	Clozapine	1.71 (1.61, 1.81)*	1.70 (354.65)	1.70 (1.62)	0.76 (−0.90)
20	Vedolizumab	1.66 (1.56, 1.78)*	1.66 (241.48)	1.66 (1.57)	0.73 (−0.94)
21	Exenatide	1.57 (1.49, 1.66)*	1.57 (264.54)	1.56 (1.49)	0.64 (−1.02)
22	Dimethyl fumarate^#^	1.54 (1.46, 1.62)*	1.54 (281.35)	1.53 (1.47)	0.61 (−1.05)
23	Bevacizumab	1.51 (1.41, 1.61)*	1.50 (152.30)	1.50 (1.42)	0.59 (−1.08)
24	Duloxetine hydrochloride	1.28 (1.20, 1.36)*	1.27 (56.59)	1.27 (1.21)	0.35 (−1.32)
25	Alendronate sodium	1.23 (1.16, 1.31)*	1.23 (49.71)	1.23 (1.17)	0.30 (−1.37)
26	Oxycodone	1.07 (1.02, 1.13)*	1.07 (7.06)	1.07 (1.03)	0.10 (−1.57)
27	Pregabalin	0.87 (0.82, 0.92)	0.87 (20.58)	0.87 (0.83)	−0.20 (−1.87)
28	Adalimumab	0.62 (0.60, 0.64)	0.63 (821.40)	0.63 (0.62)	−0.66 (−2.33)
29	Infliximab	0.52 (0.48, 0.55)	0.52 (407.56)	0.52 (0.49)	−0.94 (−2.61)
30	Etanercept	0.22 (0.21, 0.23)	0.22 (2,755.52)	0.23 (0.21)	−2.15 (−3.81)

CI, the confidence interval; EBGM, the empirical Bayes geometric mean; EBGM05, the lower limit of 95% CI, of EBGM; IC, the information component; IC025, the lower limit of 95% CI, of the IC; PRR, the proportional reporting ratio; ROR, the reporting odds ratio. The asterisk (*) means statistically significant association, i.e., the adverse events are detected as signals. The number sign (#) indicates that the risk of constipation was not documented for the FDA-approved drugs labels.

### 3.4 Time-to-onset analysis

The TTO analysis for the 26 drugs that generated valid signals was conducted. The results are presented in Table 4. The median TTO and interquartile range (IQR) for sevelamer carbonate, patiromer, solifenacin succinate, orlistat, erenumab, niraparib, semaglutide, nintedanib, cabozantinib, lenalidomide, pomalidomide were 9 (2.5, 106.25), 26 (6.75, 107.25), 11 (3, 47.75), 3 (2, 9), 31 (7, 90.75), 37 (12, 136), 29 (7, 72), 28 (6, 98.25), 29 (14, 67), 43 (14, 170), 35 (12, 224), 47 (17, 146), 11 (4, 33), 73 (20, 250.5), 7 (2, 33.75), 38 (9, 148.5), 44 (7, 528), 28 (9, 70), 1,065 (39, 3,601), 181 (51, 458.5), 31 (8, 115.25), 21 (7, 90), 49 (16, 144), 52 (12, 142.75), 251 (62, 730.5), and 31 (8, 397) days, respectively. In the assessment of WSP analysis, all shape parameters β and their 95% CI upper limits were found to be < 1. This indicates that the occurrence rate of constipation for these drugs decreases over time, suggesting an early failure type.

**TABLE 4:** Analysis of constipation time-to-onset for 26 drugs generating risk signals.

Medication	n	TTO (days)	Weibull distribution		Failure type
		Median (IQR)	Scale parameter: α (95% CI)	Shape parameter: β (95% CI)	
Sevelamer carbonate	8	9 (2.5, 106.25)	31.6 (−7.61, 70.81)	0.59 (0.27, 0.91)	Early failure
Patiromer	126	26 (6.75, 107.25)	74.67 (49.36, 99.97)	0.55 (0.48, 0.62)	Early failure
Solifenacin succinate	124	11 (3, 47.75)	34.61 (21.53, 47.69)	0.49 (0.43, 0.56)	Early failure
Orlistat	380	3 (2, 9)	9.21 (7.43, 11)	0.55 (0.52, 0.59)	Early failure
Erenumab	280	31 (7, 90.75)	56.46 (45.8, 67.12)	0.66 (0.6, 0.71)	Early failure
Niraparib	625	37 (12, 136)	85.35 (74.72, 95.98)	0.67 (0.63, 0.71)	Early failure
Semaglutide	171	29 (7, 72)	49.59 (37.83, 61.35)	0.67 (0.59, 0.74)	Early failure
Nintedanib	382	28 (6, 98.25)	69.51 (56.08, 82.95)	0.55 (0.51, 0.59)	Early failure
Cabozantinib	219	29 (14, 67)	54.99 (44.93, 65.05)	0.77 (0.69, 0.84)	Early failure
Lenalidomide	1,216	43 (14, 170)	110.28 (99.3, 121.26)	0.6 (0.57, 0.62)	Early failure
Pomalidomide	215	35 (12, 224)	109.44 (82.5, 136.39)	0.58 (0.52, 0.63)	Early failure
Enzalutamide	281	47 (17, 146)	102.33 (82.53, 122.13)	0.64 (0.59, 0.69)	Early failure
Varenicline	358	11 (4, 33)	29.88 (24.35, 35.4)	0.6 (0.55, 0.64)	Early failure
Palbociclib	304	73 (20, 250.5)	142.72 (118.47, 166.97)	0.7 (0.64, 0.76)	Early failure
Dulaglutide	84	7 (2, 33.75)	25.26 (14.96, 35.56)	0.56 (0.47, 0.64)	Early failure
Teriparatide	469	38 (9, 148.5)	81.19 (69.19, 93.19)	0.65 (0.6, 0.69)	Early failure
Dalfampridine	115	44 (7, 528)	174.04 (106.44, 241.63)	0.5 (0.43, 0.57)	Early failure
Capecitabine	182	28 (9, 70)	53.39 (41.79, 64.99)	0.71 (0.63, 0.79)	Early failure
Clozapine	399	1,065 (39, 3,601)	1,286.54 (1,039.55, 1,533.53)	0.54 (0.49, 0.58)	Early failure
Vedolizumab	450	181 (51, 458.5)	289.25 (252.81, 325.69)	0.77 (0.72, 0.83)	Early failure
Exenatide	390	31 (8, 115.25)	71.34 (59.19, 83.5)	0.62 (0.57, 0.66)	Early failure
Dimethyl fumarate	291	21 (7, 90)	70.25 (53.7, 86.81)	0.52 (0.47, 0.56)	Early failure
Bevacizumab	321	49 (16, 144)	90.11 (74.59, 105.62)	0.67 (0.62, 0.73)	Early failure
Duloxetine hydrochloride	156	52 (12, 142.75)	110.88 (79.15, 142.61)	0.58 (0.52, 0.65)	Early failure
Alendronate sodium	309	251 (62, 730.5)	418.23 (349.2, 487.25)	0.71 (0.65, 0.77)	Early failure
Oxycodone	141	31 (8, 397)	174.66 (107.94, 241.39)	0.46 (0.4, 0.52)	Early failure

n, number of cases with available time-to-onset; IQR, interquartile range; TTO, Time-to-onset.

## 4 Discussion

Constipation is a highly prevalent condition that affects individuals across all age groups, posing significant risks to human health. It may manifest independently or as a secondary symptom following the administration of medications for underlying conditions. Considering the severity of DIC, it may restrict the effective dosage of medications, consequently impacting treatment efficacy, exacerbating the progression of underlying conditions, and even potentially worsening the underlying disease state due to DIC itself (Schoenmakers et al., 2017; Hida et al., 2023). Therefore, adequate attention and early intervention should be given to DIC.

This project comprehensively evaluates the adverse reactions of DIC in the real-world setting based on the FAERS database. This study demonstrates a gradual increase in reported constipation-related cases from 2004 to 2023. The reasons for this trend may be multifactorial. Firstly, with the rising prevalence of chronic diseases and the exacerbation of population aging, there is a gradual increase in the demand for medications. Many drugs, such as antidepressants (Jeong et al., 2022), opioid medications (Liu and Brenner, 2022), anticancer drugs (Calsina-Berna et al., 2023), among others, have the potential to induce constipation. Secondly, there is an increasing awareness among individuals regarding the potential side effects of medications, leading to a deeper understanding of the adverse effects that drugs may induce and a heightened attention to the risks associated with medication use. Thirdly, enhancing reporting systems and strengthening regulatory mechanisms have facilitated more timely and accurate reporting of adverse effects of medication (Jiang et al., 2022). Analysis of percentage trends shows little change in constipation cases among all ADEs from year to year. This is related to the fact that the total number of ADEs is increasing every year as the global population increases, access to medications increases, and awareness of reporting ADEs increases. The quarterly analysis results indicate that the number of constipation cases remains relatively stable each quarter. Additionally, the percentage of constipation cases among all ADEs exhibits slight variation each quarter. This suggests that reports of constipation and all ADEs are less affected by quarterly bias.

Previous research has extensively explored age and gender differences among populations affected by constipation (Dzierżanowski and Ciałkowska-Rysz, 2018; Inkaya and Tuzer, 2020; Werth and Christopher, 2021; Ueberall et al., 2022). However, there is a lack of systematic studies investigating the characteristics of populations with DIC. We observed that DIC is more prevalent among elderly individuals and females in the adverse event cases reported to the FDA, consistent with the characteristics of the primary constipation population. Given their heightened susceptibility to constipation, clinicians should pay particular attention when prescribing medications to elderly individuals or females.

In this study, we identified the drugs associated with DIC systematically reported in the FAERS from its inception in 2004 until the third quarter of 2023. To the best of our knowledge, we are the first to utilize the FAERS database to compile a list of drugs most likely to lead to constipation adverse reactions. We found that lenalidomide, adalimumab, niraparib, erenumab, sevelamer carbonate, teriparatide, palbociclib, patiromer, varenicline, orlistat, pomalidomide, oxycodone, dimethyl fumarate, enzalutamide, exenatide, cabozantinib, clozapine, semaglutide, nintedanib, solifenacin succinate, alendronate sodium, dalfampridine, dulaglutide, pregabalin, etanercept, duloxetine hydrochloride, capecitabine, vedolizumab, infliximab, bevacizumab are the most commonly 30 drugs associated with constipation adverse reactions. Following signal detection, 26 drugs were identified as having a potential correlation with the occurrence of constipation risk, while 4 drugs did not exhibit any detected risk signals. In most cases, clinical studies, case reports, or FDA-approved drug labels have supported the association between the drugs we identified and constipation. However, previous research has yet to detail the relative frequency and risk signals of their constipation adverse reactions extensively, which we have supplemented in this work. Furthermore, after calculating the annual percentage of constipation reports among ADEs for 30 drugs, we found that 13 drugs exhibited percentages exceeding 2% in specific years. Interestingly, among these 13 drugs, 11 are ranked within the top 11 risk signals in Table 3, which enhances the reliability of our signal detection results.

Lenalidomide, with the most reported constipation events, exhibits immunomodulatory, anti-angiogenic, and anticancer properties, akin to thalidomide (Tachita et al., 2024; Onyshchenko et al., 2024). It is widely utilized in the treatment of various conditions, including multiple myeloma (Abdulkarim et al., 2020), non-Hodgkin lymphoma (Blair, 2020), and myelodysplastic syndromes (Abdallah et al., 2024). Multiple studies have reported the occurrence of constipation induced by lenalidomide (Merz et al., 2020; Witzig et al., 2009; Figaro et al., 2011). In the studies by Morschhauser F (Morschhauser et al., 2018), Leonard JP Leonard et al. (2019), Selle F Selle et al. (2014), among others (Kim et al., 2014; Wei et al., 2015), constipation consistently ranks among the top three non-hematologic events. The specific mechanism by which lenalidomide induces constipation remains unclear, necessitating further investigation.

To some extent, the more actively adverse reaction signals are observed for a drug, the more attention should be paid to its risk of causing constipation (Li et al., 2024a). Sevelamer carbonate has been identified as the drug with the strongest risk signal in our findings. It also represents the drug with the highest annual percentage of constipation reports among all ADEs for the 30 drugs analyzed. As a non-metal, calcium-free phosphate binder, it is nearly 100% excreted via feces, thereby not imposing an additional burden on the kidneys. It is primarily used to treat hyperphosphatemia in adult patients with CKD (Chen et al., 2023; Nain et al., 2023). Several studies involving sevelamer carbonate have reported the occurrence of constipation as an adverse reaction, with some cases progressing to fecal impaction (Nambiar et al., 2018; Wang et al., 2022; Wang et al., 2023). One potential reason for constipation associated with this drug is its adsorption of bile acids, which regulate colonic secretion and motility, thereby altering colonic transit (Nakaki et al., 2013; Misawa et al., 2020). Another cause might be its effect on the gut microbiota, leading to constipation (Wang et al., 2022).

Orlistat, nintedanib, palbociclib, and dimethyl fumarate are drugs identified in our study that have not been associated with constipation as an adverse effect in their FDA-approved drug labels. Orlistat, a digestive tract and metabolic drug, is primarily used to treat obesity (Feng et al., 2023). Currently, many anti-obesity drugs have been withdrawn from the market due to their association with increased risks of cardiovascular diseases, neurological disorders, and cancer. Notable examples include amphetamine, aminorex, fenfluramine/dexfenfluramine, rimonabant, sibutramine, and lorcaserin (Coulter et al., 2018; Sharretts et al., 2020; Tak and Lee, 2021). In contrast, the adverse effects of orlistat are relatively manageable, suggesting a broader potential for clinical application. Its typical side effects are primarily gastrointestinal, including oily spotting, flatus with discharge, fecal urgency, fatty/oily stool, oily evacuation, increased defecation, and fecal incontinence (Shirai et al., 2019; Syed et al., 2020). These effects are related to orlistat’s therapeutic mechanism, which involves inhibiting the absorption of dietary fats, leading to their excretion in the feces. However, the majority of studies have not reported constipation as an adverse reaction, with some even suggesting orlistat as a potential treatment for constipation (Chukhin et al., 2013; Iqbal et al., 2016). However, comprehensive evidence supporting this claim is lacking. Our study findings revealed 1918 reported cases of constipation among individuals using the medication, and all four signal detection algorithms generated risk signals, indicating a potential association between orlistat and constipation occurrence. The study by Packard et al. (2002) corroborates our findings and evaluates the causal relationship between orlistat and constipation using the Naranjo probability scale (Naranjo et al., 1981), suggesting a potential association between the two. It is speculated that the occurrence of constipation may be related to orlistat-induced diarrhea and subsequent dehydration. However, the precise pathophysiological mechanisms remain unclear. Further research is warranted to validate the causal relationship between the medication and constipation.

Nintedanib is a small molecule tyrosine kinase inhibitor with multiple targets, exerting anti-fibrotic and anti-inflammatory effects by inhibiting specific kinases, including vascular endothelial growth factor receptors (VEGFR), fibroblast growth factor receptors (FGFR), and platelet-derived growth factor receptors (PDGFR). Typically used in the treatment of fibrosing interstitial lung diseases (Di Battista et al., 2023), nintedanib has also received approval from the European Medicines Agency (EMA) for its use in combination with docetaxel for the treatment of locally metastatic or advanced adenocarcinoma-type non-small cell lung cancer (NSCLC) following first-line chemotherapy (Reck et al., 2014; Hilberg et al., 2018). The occurrence of gastrointestinal reactions appears inevitable when using VEGFR tyrosine kinase inhibitors. However, their manifestation may not be directly attributable to VEGFR inhibition but could be induced by the drug’s bystander effects on other receptors (Roodhart et al., 2008; Abdel-Rahman et al., 2016). Common gastrointestinal adverse reactions associated with nintedanib include diarrhea, nausea, abdominal pain, and vomiting (Wells et al., 2020; Di Battista et al., 2023). However, there is limited reporting on the association between nintedanib and constipation. This study identified 1,177 reported cases of constipation among individuals using nintedanib, with all four signal detection methods generating risk signals. The study by Menglin He et al. (2024) corroborates our findings, as they discovered constipation, asthenia, and flatulence associated with nintedanib, ADRs previously unknown or underestimated.

Palbociclib, the CDK4/6 targeted inhibitor, represents a significant milestone in the pharmacological landscape as the first globally approved agent for the treatment of HR+/HER2-locally advanced or metastatic breast cancer. Adverse gastrointestinal reactions associated with palbociclib predominantly comprise nausea and diarrhea (Grande et al., 2020; Sedrak et al., 2024). However, studies investigating its potential link to constipation are relatively scarce. We identified 2,127 reported occurrences of constipation among individuals utilizing this medication, prompting the generation of risk signals by three distinct signal detection algorithms. In the FLIPPER trial, the combination of palbociclib and fulvestrant demonstrated a potential for constipation compared to the placebo/fulvestrant cohort in postmenopausal women with HR+/HER2-advanced breast cancer (Tibau et al., 2023). Pu et al. (2024) conducted a meta-analysis concerning adverse events associated with CDK4/6 inhibitors in the treatment of HR+/HER2-advanced breast cancer, conclusively demonstrating a significant increase in adverse events related to constipation attributable to CDK4/6 inhibition.

Dimethyl fumarate has garnered approval from the FDA for treating relapsing-remitting multiple sclerosis and psoriasis (Manai et al., 2023; Abolfazli et al., 2023; Alwithenani et al., 2024). Common gastrointestinal adverse reactions associated with the use of dimethyl fumarate include diarrhea, abdominal pain, and nausea (Bevilacqua Rolfsen Ferreira da Silva et al., 2022; Borghi et al., 2023). O’Gorman et al. (2015) addressed the association between dimethyl fumarate and constipation, indicating a substantial disparity in the incidence rates between the placebo and dimethyl fumarate groups. Our study identified 1,517 reported cases of constipation among individuals using this medication, prompting the generation of a risk signal by one signal detection algorithm. The underlying mechanism is postulated to potentially involve the stimulation of the intestines by a metabolite of dimethyl fumarate, methanol (Palte et al., 2019; Manai et al., 2023), or it may be associated with dysbiosis in the gut microbiota (Ferri et al., 2023).

We categorized 30 medications, among which antineoplastic and immunomodulating agents (including lenalidomide, adalimumab, niraparib, palbociclib, pomalidomide, enzalutamide, cabozantinib, nintedanib, etanercept, capecitabine, vedolizumab, infliximab, and bevacizumab) emerged as the most prevalent class associated with inducing constipation. These drugs primarily involve three categories: immunosuppressants, antineoplastic agents, and endocrine therapy. For patients undergoing immunosuppressive therapy, there exists a proportional increase in gastrointestinal symptoms with a prolonged duration of treatment and improved compliance levels. These symptoms significantly impact patients’ quality of life and prognosis, particularly posing substantial risks for kidney transplant recipients (Bulbuloglu et al., 2022). Numerous studies have documented the association between immunosuppressive agents and constipation (Tong et al., 2015; Bulbuloglu et al., 2022; Costa et al., 2022). This correlation may stem from various pathways influenced by the application of immunosuppressants, including effects on intestinal function (Melenovsky et al., 2018), water absorption (Patel et al., 2017; Severin and Torres, 2019), and the gut microbiota (Gabarre et al., 2022; Low et al., 2022; Salvadori and Rosso, 2024). The mechanisms by which antineoplastic agents induce constipation remain incompletely understood (Calsina-Berna et al., 2023). The mechanisms of action differ among various drugs. For instance, platinum-based drugs may induce constipation through platinum accumulation in enteric neurons and/or chemotherapy-induced immune modulation, leading to aberrant neuro-immune interactions or collateral damage to neurons that affect gastrointestinal function (Stojanovska et al., 2015). Vincristine can cause constipation by inducing injury to enteric neurons, inhibiting gastrointestinal motility (Li et al., 2024b), or reducing transient receptor potential vanilloid 1 (TRPV1), thereby decreasing mesenteric afferent sensitivity (Li et al., 2024c). Tyrosine kinase inhibitors (TKIs) may induce constipation by interfering with normal gastrointestinal motility through the blockade of specific signal transduction pathways (Thomson et al., 2024). Furthermore, a meta-analysis has highlighted that the integrity of enteric neurons plays a significant role in constipation during antineoplastic therapy (Calsina-Berna et al., 2023). Endocrine therapy is a treatment modality primarily used for certain hormone-sensitive cancers, such as breast and prostate cancer. Relevant studies have confirmed that endocrine therapies, including anti-androgens (Akaza et al., 2016; Shore et al., 2016), anti-estrogens (Barros et al., 2023), aromatase inhibitors (Hurvitz et al., 2020), and LHRH antagonists (Ozono et al., 2018), can all lead to the occurrence of constipation.

Additionally, we analyzed the TTO of DIC. We observed that the median TTO for orlistat was 3 days, the shortest among all drugs studied. In contrast, clozapine exhibited a median TTO of 1,065 days, which was the longest among all drugs analyzed. The association between orlistat and constipation remains contentious (Chukhin et al., 2013; Iqbal et al., 2016), with a current dearth of data analysis regarding the TTO for this relationship. Chougule et al. (2018) demonstrated a median TTO of constipation episodes in patients treated with clozapine to be 60 days, which differs from our study findings. This discrepancy may be attributed to their analysis involving data from only 28 patients, indicating a relatively smaller sample size. This study utilized the largest database, FAERS, potentially offering valuable insights. The results of the WSP analysis indicate that all cases of DIC are deemed to be of the early failure type. Over time, the incidence of DIC gradually decreases. This underscores the necessity to be fully attentive to the signs of constipation that may emerge early in treatment with these medications.

Our study has several limitations. Firstly, considering FAERS as a spontaneous reporting system, issues such as underreporting, misreporting, and incomplete reporting are inevitable, potentially introducing biases to the conclusions. Secondly, we calculated only the annual percentage of constipation reports among the ADEs for each drug. Due to the lack of an exact denominator for the drug-exposed population, we were unable to estimate the actual incidence rate of DIC for each drug. Thirdly, we identified the five countries with the most constipation cases. Due to inconsistencies in awareness and concern across countries, differences in population size and the base number of medication users, significant reporting biases may exist. Finally, the significant signals we identified do not substantiate a direct causal relationship between the medications and constipation but rather present a hypothesis requiring further evaluation. However, the FAERS database remains a vital tool for pharmacovigilance analysis.

## 5 Conclusion

From reports in the FAERS database spanning from the first quarter of 2004 to the third quarter of 2023, we extracted the 30 most commonly associated drugs with DIC and their respective signal values. Among them, four drugs (orlistat, nintedanib, palbociclib, dimethyl fumarate) did not list constipation as an adverse reaction in their prescribing information. Considering that DIC may lead to poor compliance with the primary medication, physicians should be aware of the potential for DIC when prescribing causative drugs. Our list may serve to inform clinicians about some alternative medications to consider when managing secondary causes of constipation or caring for patients prone to DIC, thereby reducing the incidence and mortality associated with DIC.

## Data Availability

This data can be found here: https://fis.fda.gov/extensions/FPD-QDE-FAERS/FPD-QDE-FAERS.html, further inquiries can be directed to the corresponding author.
