# Supplementary material for: Exploring the top 30 drugs associated with drug-induced constipation based on the FDA adverse event reporting system
Source: Front Pharmacol. 2024 Sep 2;15:1443555. doi: 10.3389/fphar.2024.1443555 (PMC11402663; doi:10.3389/fphar.2024.1443555)
Supplement: Supplementary file 1 [file DataSheet1.ZIP › Supplementary material/Table S1.docx]

**Table S1.** 2×2 table for signal detection.

| Type of drug | Target adverse reaction reports | Other adverse reaction reports | Sum |
| --- | --- | --- | --- |
| Target drug | a | b | a+b |
| Other drugs | c | d | c+d |
| Sum | a+c | b+d | N=a+b+c+d |

a, number of reports containing both the target drug and target adverse reaction reports; b, number of reports containing other adverse reaction reports of the target drug; c, number of reports containing the target adverse reaction reports of other drugs; d, number of reports containing other drugs and other adverse reaction reports; N, the number of reports.
