# Supplementary material for: Exploring the top 30 drugs associated with drug-induced constipation based on the FDA adverse event reporting system
Source: Front Pharmacol. 2024 Sep 2;15:1443555. doi: 10.3389/fphar.2024.1443555 (PMC11402663; doi:10.3389/fphar.2024.1443555)
Supplement: Supplementary file 1 [file DataSheet1.ZIP › Supplementary material/Table S2.docx]

**Table S2.** Four major algorithms used for signal detection.

| Algorithms | Equation | Criteria |
| --- | --- | --- |
| ROR | ROR= (a/c)/(b/d) | 95% CI>1, N≥3 |
|  | 95%CI=e^ln(ROR)±1.96(1/a+1/b+1/c+1/d)^0.5^ |  |
| PRR | PRR=[a/(a+b)]/[c/(c+d)] | PRR≥2, χ^2^≥4, N≥3 |
|  | χ^2^=[(ad-bc)^2](a+b+c+d)/[(a+b)(c+d)(a+c)(b+d)] |  |
| MGPS | EBGM=a(a+b+c+d)/[(a+c)(a+b)] | EBGM05>2 |
|  | EBGM05=e^ln(EBGM)-1.64(1/a+1/b+1/c+1/d)^0.5^ |  |
| BCPNN | IC=log_2_a(a+b+c+d)/[(a+c)(a+b)] | IC025>0 |
|  | IC025 = e^ln(IC) − 1.96(1/a + 1/b + 1/c + 1/d)^0.5^ |  |

a, number of reports containing both the target drug and target adverse reaction reports; b, number of reports containing other adverse reaction reports of the target drug; c, number of reports containing the target adverse reaction reports of other drugs; d, number of reports containing other drugs and other adverse reaction reports. 95%CI, 95% confidence interval; N, the number of reports; χ^2^, chi-squared; EBGM, empirical Bayesian geometric mean; EBGM05, the lower limit of 95% CI of EBGM; IC, information component; IC025, the lower limit of 95% CI of the IC. ROR, reporting odds ratio; PRR, proportional reporting ratio; MGPS, multi-item gamma poisson shrinker; BCPNN, Bayesian confidence propagation neural network.
